# Supplementary material for: High-resolution maps show that rubber causes substantial deforestation
Source: Nature. 2023 Oct 18;623(7986):340–6. doi: 10.1038/s41586-023-06642-z (PMC10632130; doi:10.1038/s41586-023-06642-z)
Supplement: Supplementary file 1 — Supplementary Note, Tables 1–7 and Fig. 1. [file 41586_2023_6642_MOESM1_ESM.docx]

# Supplementary Information

High-resolution maps show that rubber causes substantial deforestation

## Supplementary Note | Definitions of ‘forest’ and ‘deforestation’

The definition of forest is a critical issue, for example when comparing different deforestation estimates (e.g., in Table 2). It is very difficult to distinguish plantation forest and agricultural tree crops from natural forest and studies deal with this in different ways. In addition, studies use different baseline tree cover thresholds.

On the first point, Goldman et al. (2020)^1^ opted to use the term ‘deforestation’ to mean all tree cover loss, including rotational tree crop and plantation clearing. Hurni & Fox (2018)^2^ on the other hand track changes in a range of tree crops and thereby minimise their confounding influence. Pendrill et al. (2019)^3^ took steps to exclude tree plantations in Indonesia and Malaysia, and elsewhere use ‘deforestation’ to mean all tree cover loss. As described in the Methods, in this study we minimise the erroneous inclusion of plantations by (1) tracking only the first deforestation date going back as far as the Landsat imagery allows (1993), and (2) only counting pixels above an NBR threshold (prior to the change detection) to reduce the inclusion of areas that have experienced degradation or deforestation prior to 1993.

Our deforestation estimates for Indonesia and Malaysia are higher than those of Pendrill et al. (2019)^3^. However, even if one assumed that for these two countries our data presents an overestimate (i.e. includes plantation rotation), and if one replaced our estimates for Indonesia and Malaysia with those of Pendrill et al. (2019)^3^, our overall figure for annual rubber-related deforestation would still be more than twice as high. Furthermore, even if one assumed that no deforestation took place at all in Indonesia, Malaysia and also Thailand, our annual deforestation figure would still be above the total figure provided by Pendrill et al. (2019)^3^. It is also noteworthy that although Hurni and Fox (2018)^2^ employ a strict approach for dealing with tree crops, their deforestation figures are generally in line, and in some areas (Cambodia) above, ours.

On the second point: Pendrill et al. (2022)^3^ use a stricter definition of baseline tree cover than employed in this study (using a higher canopy cover threshold of 25% versus 10% in this study); however, according to a sensitivity analysis conducted by the authors themselves a lower (10%) tree cover threshold would generally not lead to big differences in estimated deforestation (apart from in Africa where the difference is more notable)^3^. Goldman et al. (2020)^1^ use an even stricter tree cover threshold of 30%, but their deforestation figures are generally consistent with (or above) our figures; this may be because the effect of a stricter tree cover threshold is balanced by a less strict approach to plantation inclusion (Goldman et al. (2020) use a baseline of 2000 as opposed to the baseline of 1993 used in this study).

**Supplementary Tables**

**Supplementary Table 1** | **Accuracy assessment and area estimation for mapping rubber across Southeast Asia.** Accuracy and area estimation calculations follow Olofsson et al. Remote Sensing of Environment 148, 42-57 (2014). The substantially higher estimated than mapped area for rubber illustrates that our mapped area is conservative. Potential rubber omissions mainly occurred in insular Southeast Asia (Supplementary Table 3). The estimates for mainland Southeast Asia achieved higher accuracy and precision (Supplementary Table 2). For brevity we use the term ‘Forest’ in the table below, but this includes all types of tree cover other than rubber.

| **A Summary of mapped area** | | |  |
| --- | --- | --- | --- |
|  | Pixels | Area (ha) | Area proportion (Wi) |
| *Forest* | 19,784,950,116 | 197,849,501 | 0.933 |
| *Rubber* | 1,415,108,993 | 14,151,090 | 0.067 |
| Total | 21,200,059,109 | 212,000,591 | 1 |

| **B Error matrix of reference data** | | |  |  |  |
| --- | --- | --- | --- | --- | --- |
|  |  | **Reference** |  |  |  |
|  |  | Forest | Rubber | Total | User's accuracy |
| **Map** | *Forest* | 338 | 19 | 357 | 0.947 |
|  | *Rubber* | 2 | 302 | 304 | 0.993 |
|  | Total | 340 | 321 | 661 |  |
| Producer's accuracy | | 0.994 | 0.941 | **Overall** | 0.968 |

| **C Error matrix of estimated area proportions** | | | |  |  |  |  |
| --- | --- | --- | --- | --- | --- | --- | --- |
|  |  | **Reference** |  |  |  |  |  |
|  |  | Forest | Rubber | Total (Wi) | User's accuracy | *Var* | *CI* |
| **Map** | *Forest* | 0.884 | 0.050 | 0.933 | 0.947 | 0.0001 | 0.02 |
|  | *Rubber* | 0.000 | 0.066 | 0.067 | 0.993 | 0.0000 | 0.01 |
|  | Total | 0.884 | 0.116 | 1.000 |  |  |  |
| Producer's accuracy | | 1.000 | 0.572 | **Overall** | **0.950** | **0.0001** | **0.02** |
|  | *Var* | 0.0000 | 0.0030 |  |  |  |  |
|  | *CI* | 0.00 | 0.11 |  |  |  |  |

| **D Mapped and estimated area (with confidence intervals)** | | | | |
| --- | --- | --- | --- | --- |
|  | mapped area (ha) | estimated area (ha) | Lower 95% CI area (ha) | Upper 95% CI area (ha) |
| Forest | 197,849,501 | 187,412,795 | 182,797,471 | 192,028,119 |
| Rubber | 14,151,090 | 24,587,796 | 19,972,472 | 29,203,120 |
| Total | 212,000,591 | 212,000,591 |  |  |

**Supplementary Table 2** | **Subregional accuracy assessment and area estimation for mapping rubber across mainland Southeast Asia.** Accuracy and area estimation calculations follow Olofsson et al. Remote Sensing of Environment 148, 42-57 (2014). Mainland Southeast Asia includes China, Myanmar, Thailand, Laos, Vietnam and Cambodia. For brevity we use the term ‘Forest’ in the table below, but this includes all types of tree cover other than rubber.

| **A Summary of mapped area** | | |  |
| --- | --- | --- | --- |
|  | Pixels | Area (ha) | Area proportion (Wi) |
| *Forest* | 5,230,757,420 | 52,307,574 | 0.861 |
| *Rubber* | 841,983,386 | 8,419,834 | 0.139 |
| Total | 6,072,740,806 | 60,727,408 | 1 |

| **B Error matrix of reference data** | | |  |  |  |
| --- | --- | --- | --- | --- | --- |
|  |  | **Reference** |  |  |  |
|  |  | Forest | Rubber | Total | User's accuracy |
| **Map** | *Forest* | 238 | 1 | 239 | 0.996 |
|  | *Rubber* | 1 | 282 | 283 | 0.996 |
|  | Total | 239 | 283 | 522 |  |
| Producer's accuracy | | 0.996 | 0.996 | **Overall** | 0.996 |

| **C Error matrix of estimated area proportions** | | | |  |  |  |  |
| --- | --- | --- | --- | --- | --- | --- | --- |
|  |  | **Reference** |  |  |  |  |  |
|  |  | Forest | Rubber | Total (Wi) | User's accuracy | *Var* | *CI* |
| **Map** | *Forest* | 0.858 | 0.004 | 0.861 | 0.996 | 0.0000 | 0.01 |
|  | *Rubber* | 0.000 | 0.138 | 0.139 | 0.996 | 0.0000 | 0.01 |
|  | Total | 0.858 | 0.142 | 1.000 |  |  |  |
| Producer's accuracy | | 0.999 | 0.975 | **Overall** | **0.996** | **0.0000** | **0.01** |
|  | *Var* | 0.0029 | 0.0000 |  |  |  |  |
|  | *CI* | 0.11 | 0.00 |  |  |  |  |

| **D Mapped and estimated area (with confidence intervals)** | | | | |
| --- | --- | --- | --- | --- |
|  | mapped area (ha) | estimated area (ha) | Lower 95% CI area (ha) | Upper 95% CI area (ha) |
| Forest | 52,307,574 | 52,118,466 | 51,685,555 | 52,551,377 |
| Rubber | 8,419,834 | 8,608,942 | 8,176,031 | 9,041,853 |
| Total | 60,727,408 | 60,727,408 |  |  |

**Supplementary Table 3** | **Subregional accuracy assessment and area estimation for mapping rubber across insular Southeast Asia.** Accuracy and area estimation calculations follow Olofsson et al. Remote Sensing of Environment 148, 42-57 (2014). Our estimates for this insular Southeast Asia have considerably more uncertainty than for mainland Southeast Asia due to greater mapping challenges (see main manuscript Discussion and Methods) and a comparatively low number of sample points. The substantially higher estimated than mapped area for rubber illustrates that our mapped area for insular Southeast Asia could be conservative. Insular Southeast Asia includes Malaysia and Indonesia. For brevity we use the term ‘Forest’ in the table below, but this includes all types of tree cover other than rubber.

| **A Summary of mapped area** | | |  |
| --- | --- | --- | --- |
|  | Pixels | Area (ha) | Area proportion (Wi) |
| *Forest* | 14,554,192,696 | 145,541,927 | 0.962 |
| *Rubber* | 573,125,607 | 5,731,256 | 0.038 |
| Total | 15,127,318,303 | 151,273,183 | 1 |

| **B Error matrix of reference data** | | |  |  |  |
| --- | --- | --- | --- | --- | --- |
|  |  | **Reference** |  |  |  |
|  |  | Forest | Rubber | Total | User's accuracy |
| **Map** | *Forest* | 100 | 18 | 118 | 0.847 |
|  | *Rubber* | 1 | 20 | 21 | 0.952 |
|  | Total | 101 | 38 | 139 |  |
| Producer's accuracy | | 0.990 | 0.526 | **Overall** | **0.863** |

| **C Error matrix of estimated area proportions** | | | |  |  |  |  |
| --- | --- | --- | --- | --- | --- | --- | --- |
|  |  | **Reference** |  |  |  |  |  |
|  |  | Forest | Rubber | Total (Wi) | User's accuracy | *Var* | *CI* |
| **Map** | *Forest* | 0.815 | 0.147 | 0.962 | 0.847 | 0.0011 | 0.07 |
|  | *Rubber* | 0.002 | 0.036 | 0.038 | 0.952 | 0.0023 | 0.09 |
|  | Total | 0.817 | 0.183 | 1.000 |  |  |  |
| Producer's accuracy | | 0.998 | 0.197 | **Overall** | **0.851** | **0.0010** | **0.06** |
|  | *Var* | 0.0327 | 0.0008 |  |  |  |  |
|  | *CI* | 0.35 | 0.06 |  |  |  |  |

| **D Mapped and estimated area (with confidence intervals)** | | | | |
| --- | --- | --- | --- | --- |
|  | mapped area (ha) | estimated area (ha) | Lower 95% CI area (ha) | Upper 95% CI area (ha) |
| Forest | 145,541,927 | 123,613,533 | 114,116,336 | 133,110,731 |
| Rubber | 5,731,256 | 27,659,650 | 18,162,452 | 37,156,848 |
| Total | 151,273,183 | 151,273,183 |  |  |

**Supplementary Table 4** | **Accuracy assessment and area estimation for the timing of rubber-related deforestation across Southeast Asia.** Accuracy and area estimation calculations follow Olofsson et al. Remote Sensing of Environment 148, 42-57 (2014). The assessment suggests that our mapped area may underestimate deforestation up until and including 2000 and consequently overestimate deforestation after 2000. This is in line with expectations as more and improved imagery is available for recent dates. To be conservative, we report both figures for deforestation post 2000 and in the abstract concentrate on the lowest of all estimates (i.e., the lower 95% confidence interval) as recent deforestation is more policy relevant than deforestation prior to 2000. The large confidence intervals are due to a relatively small number of sample points with deforestation dates. It is also worth noting that if our mapped rubber area is conservative (Supplementary Table 1), so are our deforestation estimates as deforestation was only quantified in areas mapped as rubber.

| **A Summary of mapped area** | | |  |
| --- | --- | --- | --- |
|  | Pixels | Area (ha) | Area proportion (Wi) |
| *1993-2000* | 12,259,958 | 1,103,396 | 0.270 |
| *post 2000* | 33,108,575 | 2,979,772 | 0.730 |
| Total | 45,368,534 | 4,083,168 | 1 |

| **B Error matrix of reference data** | | |  |  |  |
| --- | --- | --- | --- | --- | --- |
|  |  | **Reference** |  |  |  |
|  |  | 1993-2000 | post 2000 | Total | User's accuracy |
| **Map** | *1993-2000* | 17 | 1 | 18 | 0.944 |
|  | *post 2000* | 9 | 40 | 49 | 0.816 |
|  | Total | 26 | 41 | 67 |  |
| Producer's accuracy | | 0.654 | 0.976 | **Overall** | **0.851** |

| **C Error matrix of estimated area proportions** | | | |  |  |  |  |
| --- | --- | --- | --- | --- | --- | --- | --- |
|  |  | **Reference** |  |  |  |  |  |
|  |  | 1993-2000 | post 2000 | Total (Wi) | User's accuracy | *Var* | *CI* |
| **Map** | *1993-2000* | 0.255 | 0.015 | 0.270 | 0.944 | 0.0031 | 0.11 |
|  | *post 2000* | 0.134 | 0.596 | 0.730 | 0.816 | 0.0031 | 0.11 |
|  | Total | 0.389 | 0.611 | 1.000 |  |  |  |
| Producer's accuracy | | 0.656 | 0.975 | **Overall** | **0.851** | **0.0019** | **0.09** |
|  | *Var* | 0.0293 | 0.0000 |  |  |  |  |
|  | *CI* | 0.34 | 0.01 |  |  |  |  |

| **D Mapped and estimated area (with confidence intervals)** | | | | |
| --- | --- | --- | --- | --- |
|  | mapped area (ha) | estimated area (ha) | Lower 95% CI area (ha) | Upper 95% CI area (ha) |
| 1993-2000 | 1,103,396 | 1,589,402 | 1,241,574 | 1,937,229 |
| post 2000 | 2,979,772 | 2,493,767 | 2,145,939 | 2,841,594 |
| Total | 4,083,168 | 4,083,168 |  |  |

**Supplementary Table 5** | **Subregional accuracy assessment and area estimation for the timing of rubber-related deforestation across mainland Southeast Asia.** Accuracy and area estimation calculations follow Olofsson et al. Remote Sensing of Environment 148, 42-57 (2014). Mainland Southeast Asia includes China, Myanmar, Thailand, Laos, Vietnam and Cambodia.

| **A Summary of mapped area** | | |  |
| --- | --- | --- | --- |
|  | Pixels | Area (ha) | Area proportion (Wi) |
| *1993-2000* | 4,704,794 | 423,431 | 0.210 |
| *post 2000* | 17,734,682 | 1,596,122 | 0.790 |
| Total | 22,439,476 | 2,019,553 | 1 |

| **B Error matrix of reference data** | | |  |  |  |
| --- | --- | --- | --- | --- | --- |
|  |  | **Reference** |  |  |  |
|  |  | 1993-2000 | post 2000 | Total | User's accuracy |
| **Map** | *1993-2000* | 10 | 1 | 11 | 0.909 |
|  | *post 2000* | 5 | 22 | 27 | 0.815 |
|  | Total | 15 | 23 | 38 |  |
| Producer's accuracy | | 0.667 | 0.957 | **Overall** | **0.842** |

| **C Error matrix of estimated area proportions** | | | |  |  |  |  |
| --- | --- | --- | --- | --- | --- | --- | --- |
|  |  | **Reference** |  |  |  |  |  |
|  |  | 1993-2000 | post 2000 | Total (Wi) | User's accuracy | *Var* | *CI* |
| **Map** | *1993-2000* | 0.191 | 0.019 | 0.210 | 0.909 | 0.0083 | 0.18 |
|  | *post 2000* | 0.146 | 0.644 | 0.790 | 0.815 | 0.0058 | 0.15 |
|  | Total | 0.337 | 0.663 | 1.000 |  |  |  |
| Producer's accuracy | | 0.566 | 0.971 | **Overall** | **0.835** | **0.0040** | **0.12** |
|  | *Var* | 0.0445 | 0.0000 |  |  |  |  |
|  | *CI* | 0.41 | 0.01 |  |  |  |  |

| **D Mapped and estimated area (with confidence intervals)** | | | | |
| --- | --- | --- | --- | --- |
|  | mapped area (ha) | estimated area (ha) | Lower 95% CI area (ha) | Upper 95% CI area (ha) |
| 1993-2000 | 423,431 | 680,516 | 430,534 | 930,497 |
| post 2000 | 1,596,122 | 1,339,037 | 1,089,056 | 1,589,019 |
| Total | 2,019,553 | 2,019,553 |  |  |

**Supplementary Table 6** | **Subregional accuracy assessment and area estimation for the timing of rubber-related deforestation across insular Southeast Asia.** Accuracy and area estimation calculations follow Olofsson et al. Remote Sensing of Environment 148, 42-57 (2014). Insular Southeast Asia includes Malaysia and Indonesia.

| **A Summary of mapped area** | | |  |
| --- | --- | --- | --- |
|  | Pixels | Area (ha) | Area proportion (Wi) |
| *1993-2000* | 7,555,165 | 679,965 | 0.330 |
| *post 2000* | 15,373,893 | 1,383,651 | 0.670 |
| Total | 22,929,058 | 2,063,615 | 1 |

| **B Error matrix of reference data** | | |  |  |  |
| --- | --- | --- | --- | --- | --- |
|  |  | **Reference** |  |  |  |
|  |  | 1993-2000 | post 2000 | Total | User's accuracy |
| **Map** | *1993-2000* | 7 | 10 | 17 | 0.412 |
|  | *post 2000* | 4 | 18 | 22 | 0.818 |
|  | Total | 11 | 28 | 39 |  |
| Producer's accuracy | | 0.636 | 0.643 | **Overall** | **0.641** |

| **C Error matrix of estimated area proportions** | | | |  |  |  |  |
| --- | --- | --- | --- | --- | --- | --- | --- |
|  |  | **Reference** |  |  |  |  |  |
|  |  | 1993-2000 | post 2000 | Total (Wi) | User's accuracy | *Var* | *CI* |
| **Map** | *1993-2000* | 0.136 | 0.194 | 0.330 | 0.412 | 0.0151 | 0.24 |
|  | *post 2000* | 0.122 | 0.549 | 0.670 | 0.818 | 0.0071 | 0.16 |
|  | Total | 0.258 | 0.742 | 1.000 |  |  |  |
| Producer's accuracy | | 0.527 | 0.739 | **Overall** | **0.684** | **0.0048** | **0.14** |
|  | *Var* | 0.0545 | 0.0004 |  |  |  |  |
|  | *CI* | 0.46 | 0.04 |  |  |  |  |

| **D Mapped and estimated area (with confidence intervals)** | | | | |
| --- | --- | --- | --- | --- |
|  | mapped area (ha) | estimated area (ha) | Lower 95% CI area (ha) | Upper 95% CI area (ha) |
| 1993-2000 | 679,965 | 531,558 | 250,511 | 812,606 |
| post 2000 | 1,383,651 | 1,532,057 | 1,251,009 | 1,813,105 |
| Total | 2,063,615 | 2,063,615 |  |  |

**Supplementary Table 7** | **Proportion of area without clear images due to cloud cover.** Total study area refers to the area of the ESA tree cover base map applied in this study.

|  | China | Myanmar | Cambodia | Vietnam | Laos | Thailand | Malaysia | Indonesia | **Total** |
| --- | --- | --- | --- | --- | --- | --- | --- | --- | --- |
| Area without clear images (A, km^2^) | 14 | 28 | 46 | 2,965 | 24 | 133 | 29,096 | 110,480 | 142,786 |
| Total study area  (B, km^2^) | 42,526 | 290,286 | 84,776 | 208,785 | 179,500 | 278,254 | 297,356 | 1,593,479 | 2,974,963 |
| % (A/B) | 0.03% | 0.01% | 0.05% | 1.42% | 0.01% | 0.05% | 9.78% | 6.93% | 4.80% |

**Supplementary Table 8** | **Variable contribution importance to the Random Forest classifier.** Variables starting with a B refer to individual Sentinel-2 bands. Others are spectral indices (for their definition and calculation see main manuscript Methods).

|  | **RVMI** | **B4** | **B11** | **MNBR** | **B6** | **NBR** | **NDWI** | **B12** | **EVI** |
| --- | --- | --- | --- | --- | --- | --- | --- | --- | --- |
| **Relative importance** | 0.0767 | 0.0667 | 0.0625 | 0.0618 | 0.0613 | 0.0611 | 0.0592 | 0.0587 | 0.0574 |
|  | **B2** | **NDVI** | **B5** | **B7** | **SAVI** | **B8A** | **B8** | **B3** |  |
| **Relative importance** | 0.0564 | 0.0562 | 0.0555 | 0.0550 | 0.0549 | 0.0543 | 0.0527 | 0.0494 |  |

**Supplementary Table 9** | **Hyperparameter settings for the Random Forest classifier and the LandTrendr algorithm.** Definitions for the Random Forest terms are available in Google Earth Engine. For LandTrendr definitions are available here: <https://emapr.github.io/LT-GEE/lt-gee-requirements.html#lt-parameters>.

| **Random Forest** | **Number of trees** | **Bag fraction** | **minLeafPopulation** | **Seed** |
| --- | --- | --- | --- | --- |
|  | 100 | 0.632 | 2 | 2 |
| **LandTrendr** | **maxSegments** | **spikeThreshold** | **vertexCountOvershoot** | **preventOneYearRecovery** |
|  | 8 | 0.9 | 3 | True |
|  | **recoveryThreshold** | **pvalThreshold** | **bestModelProportion** | **minObservationsNeeded** |
|  | 0.25 | 0.1 | 0.75 | 4 |

**Supplementary Figures**

**Supplementary Fig. 1 | Example of the visual interpretation process carried out in Collect Earth Online and Google Earth Pro.** The following images show examples of the visual interpretation process during which the interpreters classified reference ground samples by answering these questions: (1) What was the land cover in 2021? (2) If it was rubber, is there evidence of past deforestation? (3) If there is evidence for deforestation, in which year did this occur? The process was carried out by two interpreters, who had received prior training by experts. Contentious points were reviewed jointly.

In Collect Earth Online (CEO) the interpreters had access to very high-resolution imagery from BingMaps, Mapbox Satellite and Planet NICFI. In addition, the CEO GeoDash interface provided access to Sentinel-2 image composites for the rubber defoliation and refoliation windows between 2017-2021, Landsat image composites for the rubber defoliation and refoliation windows between 1988-2016, Landsat spectral indices time-series (e.g., NDVI, NDWI) between 1988-2021, and the CEO Degradation Tool.

Historical very high-resolution images (with acquisition dates) from Google Earth Pro desktop were also used to support the visual interpretation process.

Image: CEO, Mapbox Satellite.


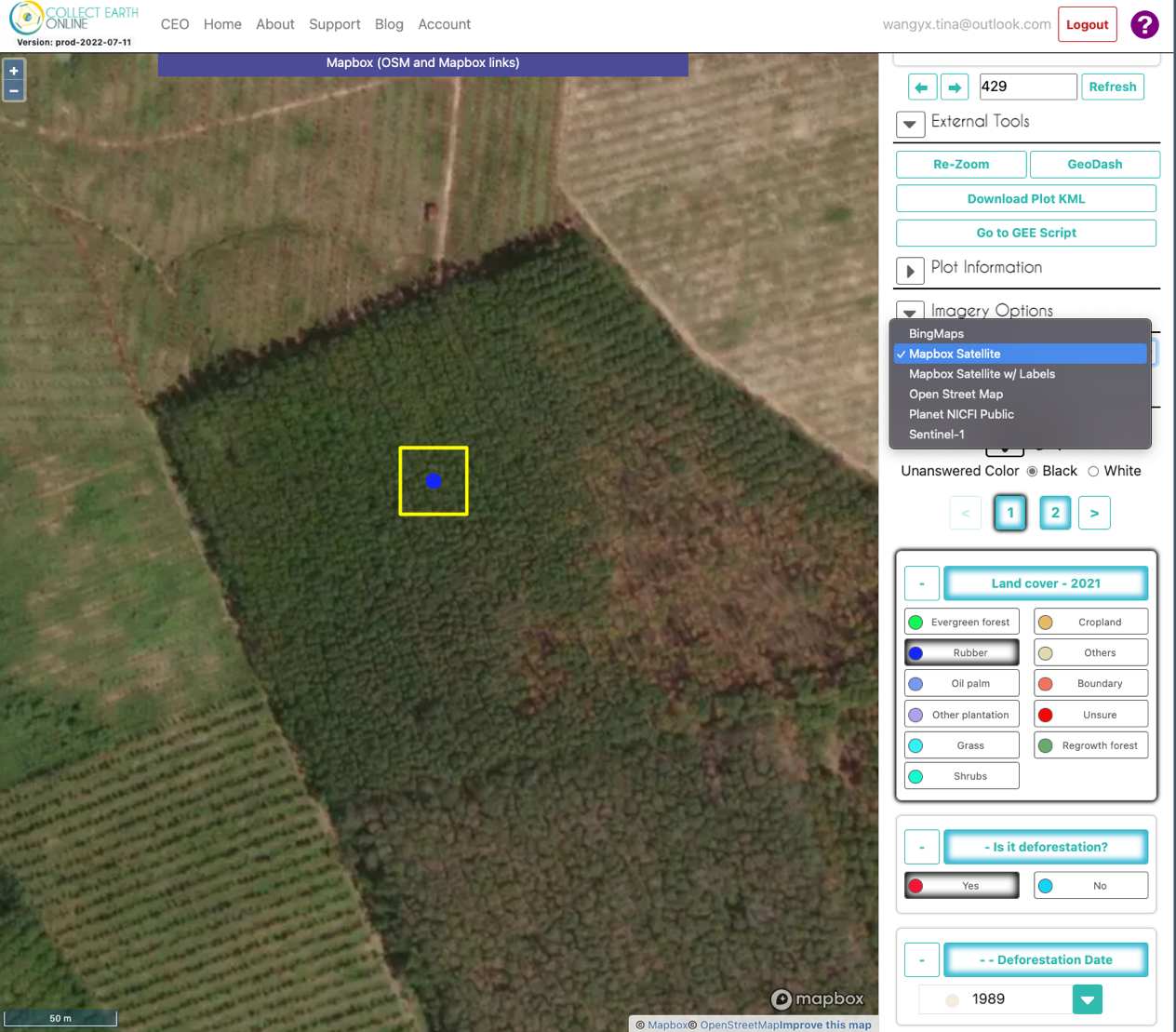


**The following images show a rubber point in southern Thailand (99.2209 longitude, 8.6305 latitude) exhibiting the characteristic phenology pattern with defoliation during January to February and refoliation during March to April. No deforestation event was detected.**

| **Monthly imagery from Planet NICFI.** Images: Collect Earth Online, Planet NICFI | | | |
| --- | --- | --- | --- |
| Planet NICFI (Jan-2022) | Planet NICFI (Feb-2022) | Planet NICFI (Mar-2022) | Planet NICFI (Apr-2022) |
| 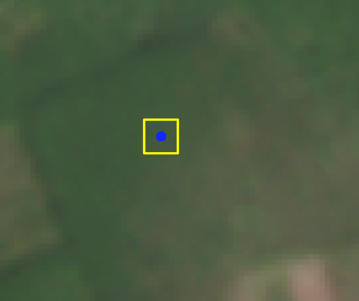 | 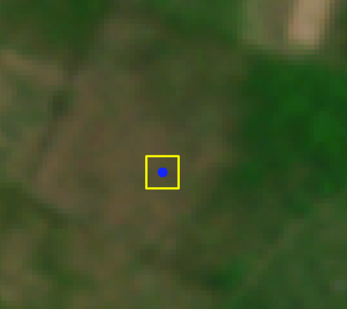 | 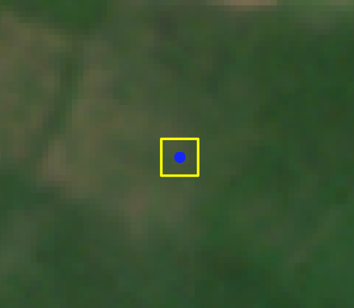 | 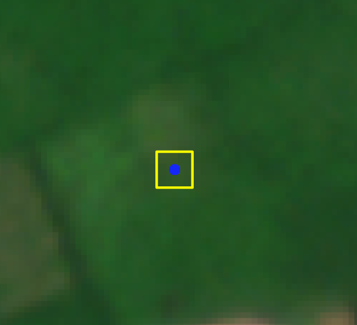 |
| Planet NICFI (Jan-2021) | Planet NICFI (Feb-2021) | Planet NICFI (Mar-2021) | Planet NICFI (Apr-2021) |
| 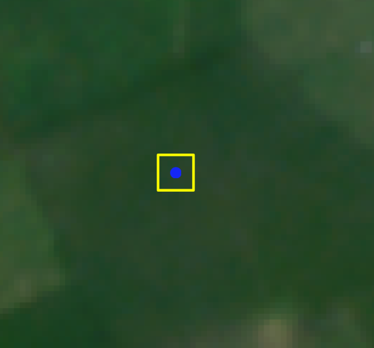 | 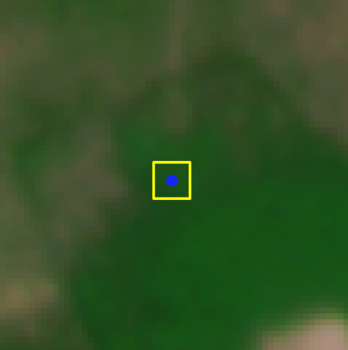 | 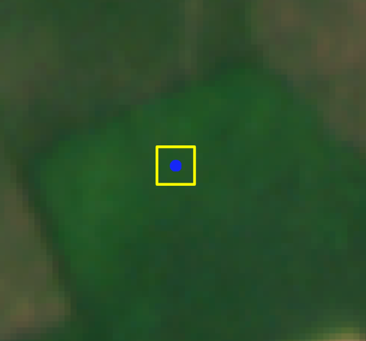 | 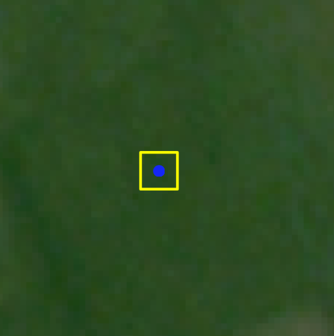 |

| **Historical very-high resolution images for this point were available for 2014, 2016, 2017, 2018, 2019 and 2020.** Images: Google, Maxar Technology, CNES/Airbus | | |
| --- | --- | --- |
| 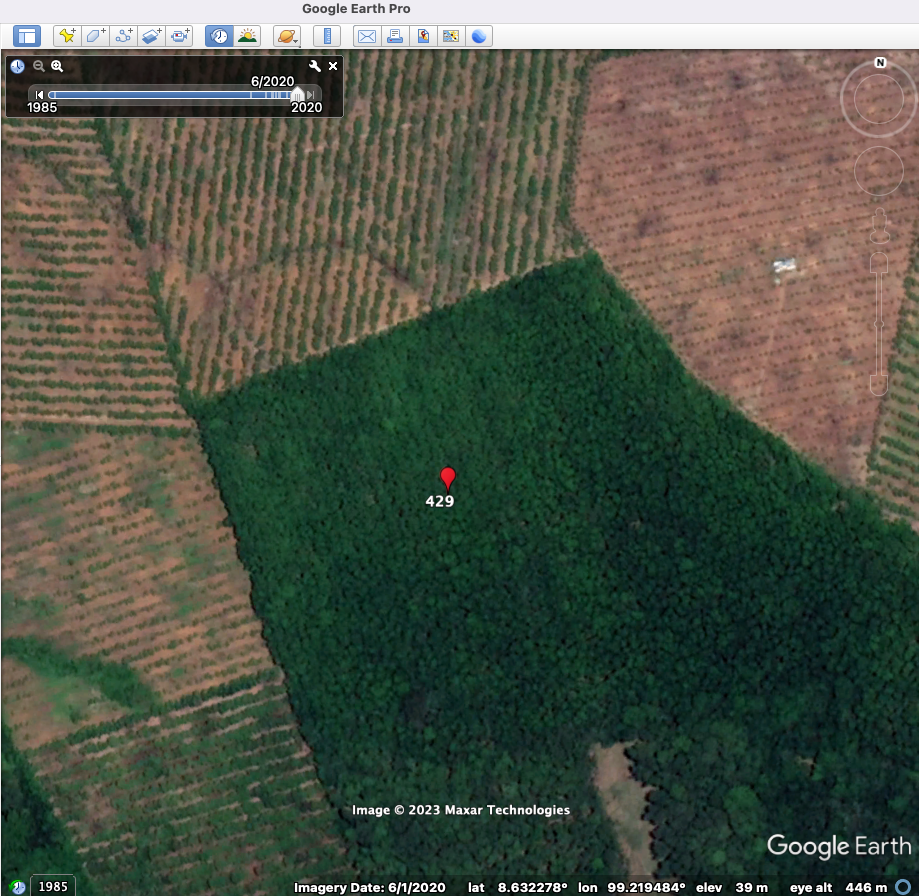 | | |
| 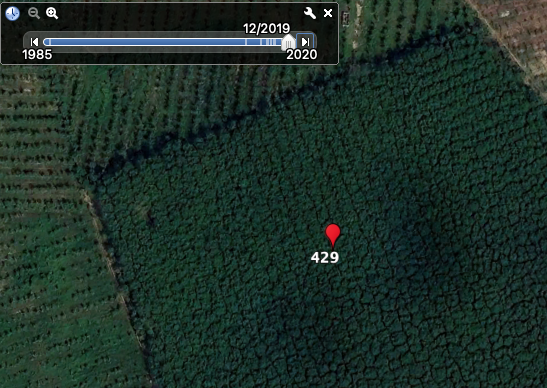  Dec 2019 | 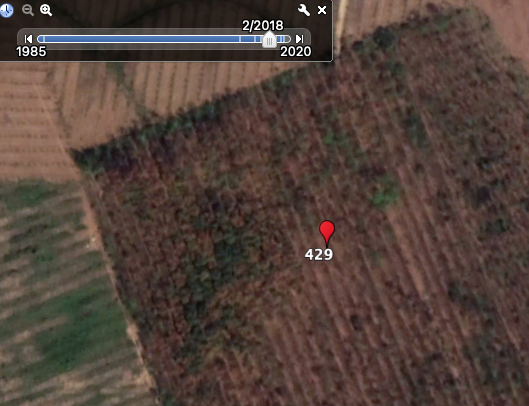  Feb 2018 | 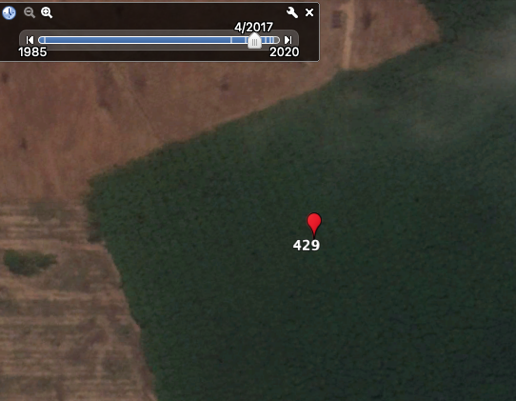  Apr 2017 |
| 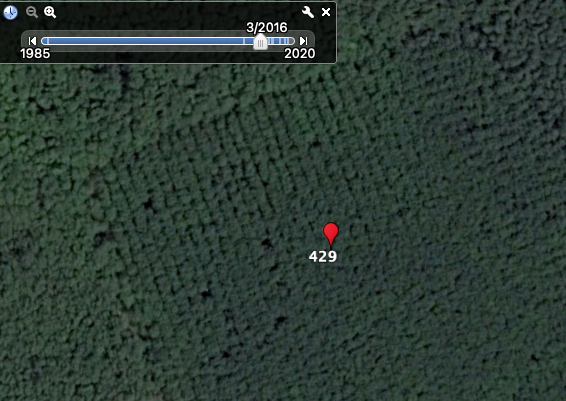  Mar 2016 | 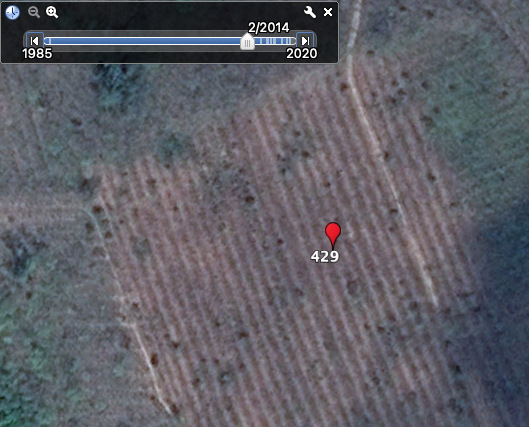  Feb 2014 |  |

**Example of the CEO Dashboard, providing interpreters with NDVI, NDWI and NDFI time series and image composites based on both Sentinel 2 and Landsat data.**

Images: CEO, ESA Sentinel 2, NASA Landsat, Mapbox Satellite

| 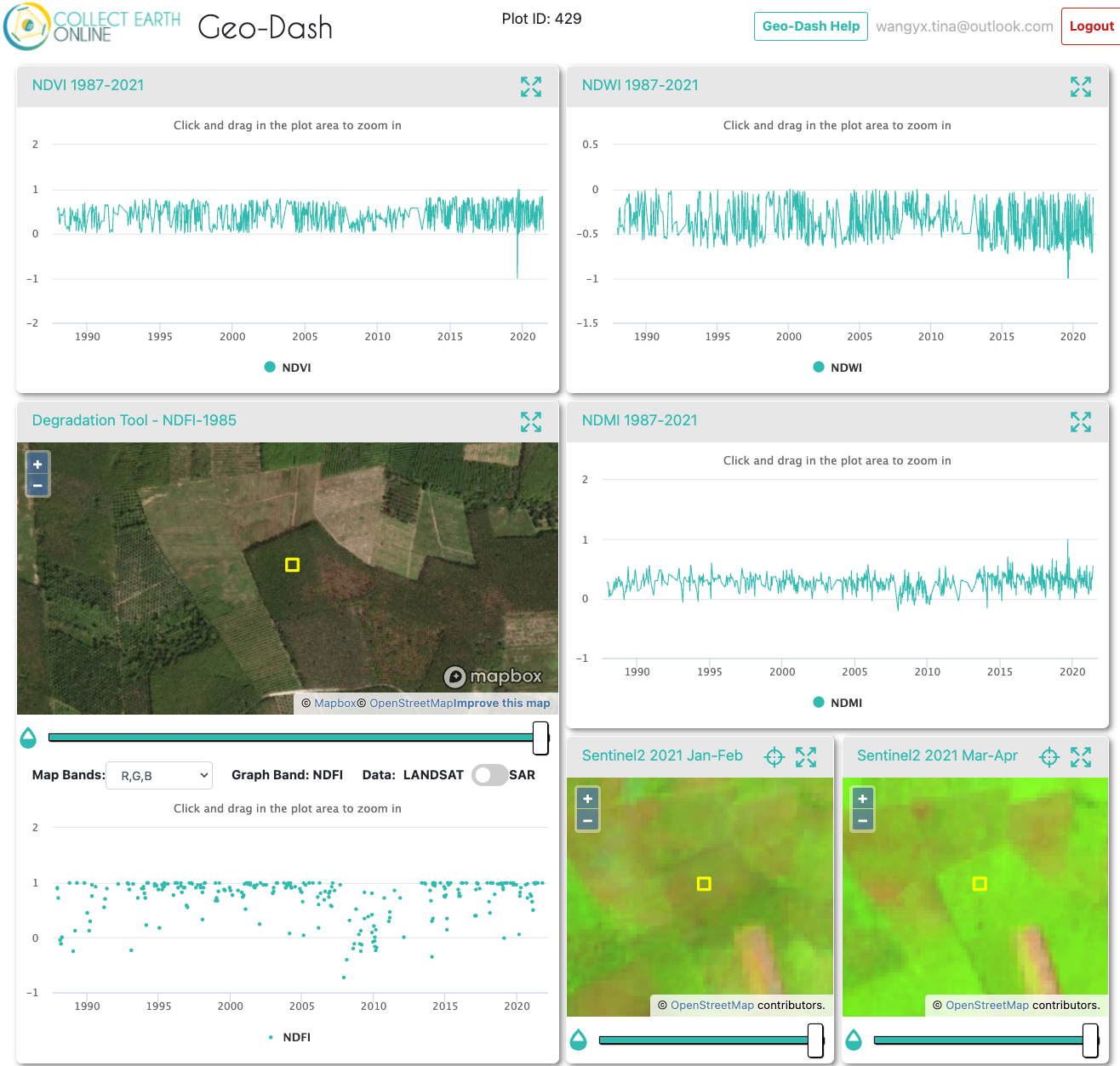 |
| --- |
| 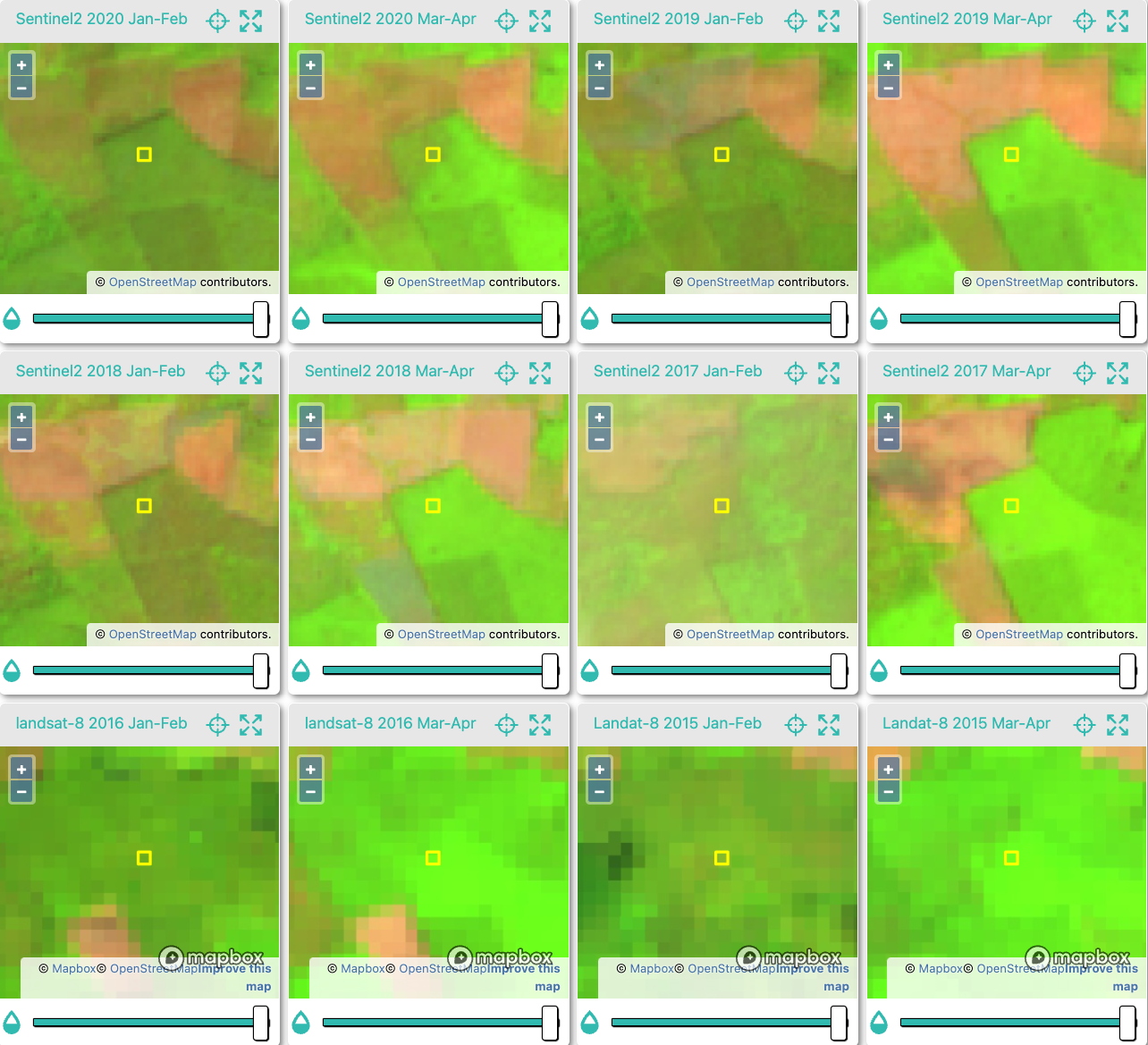 |
| 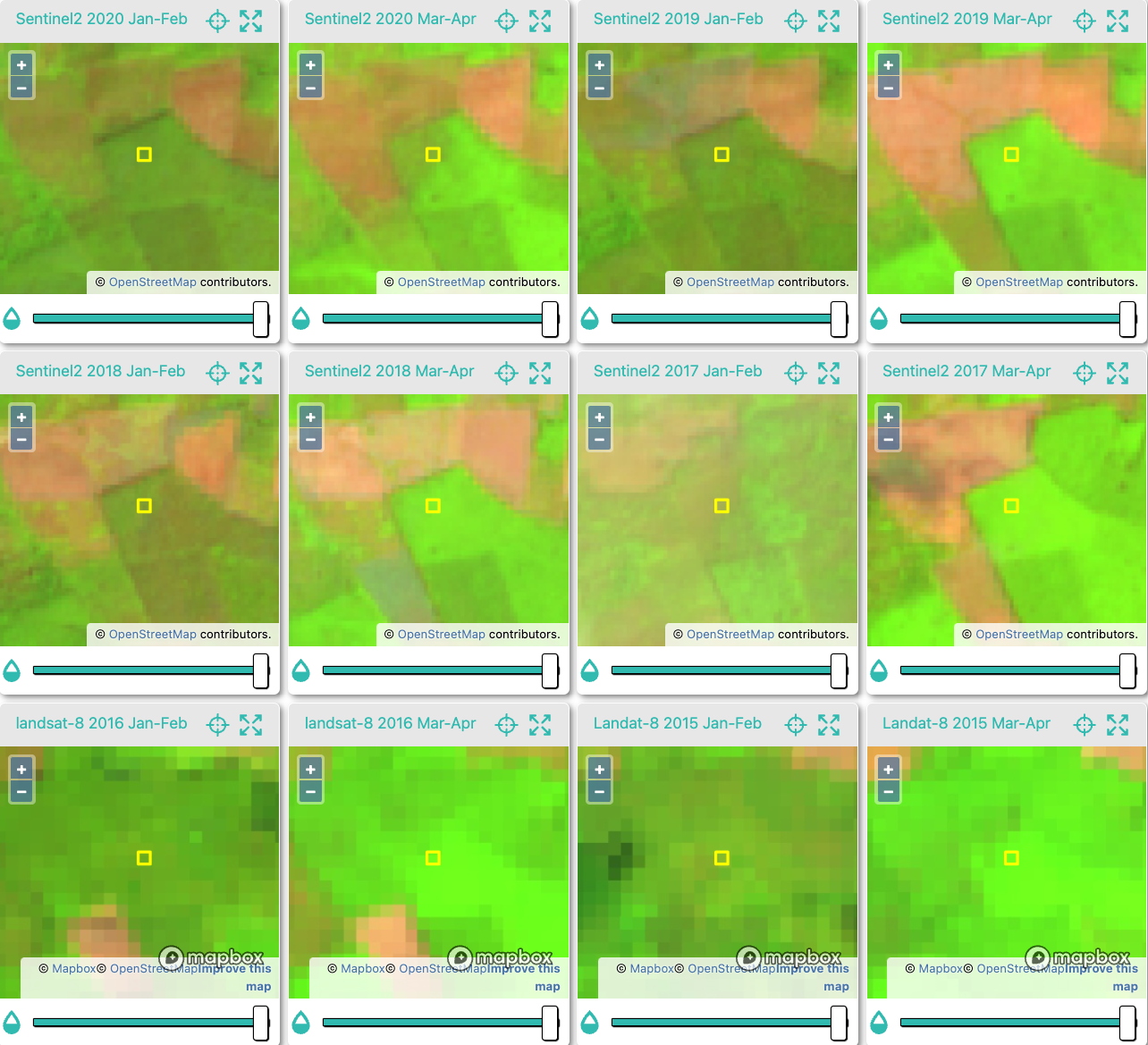 |
| 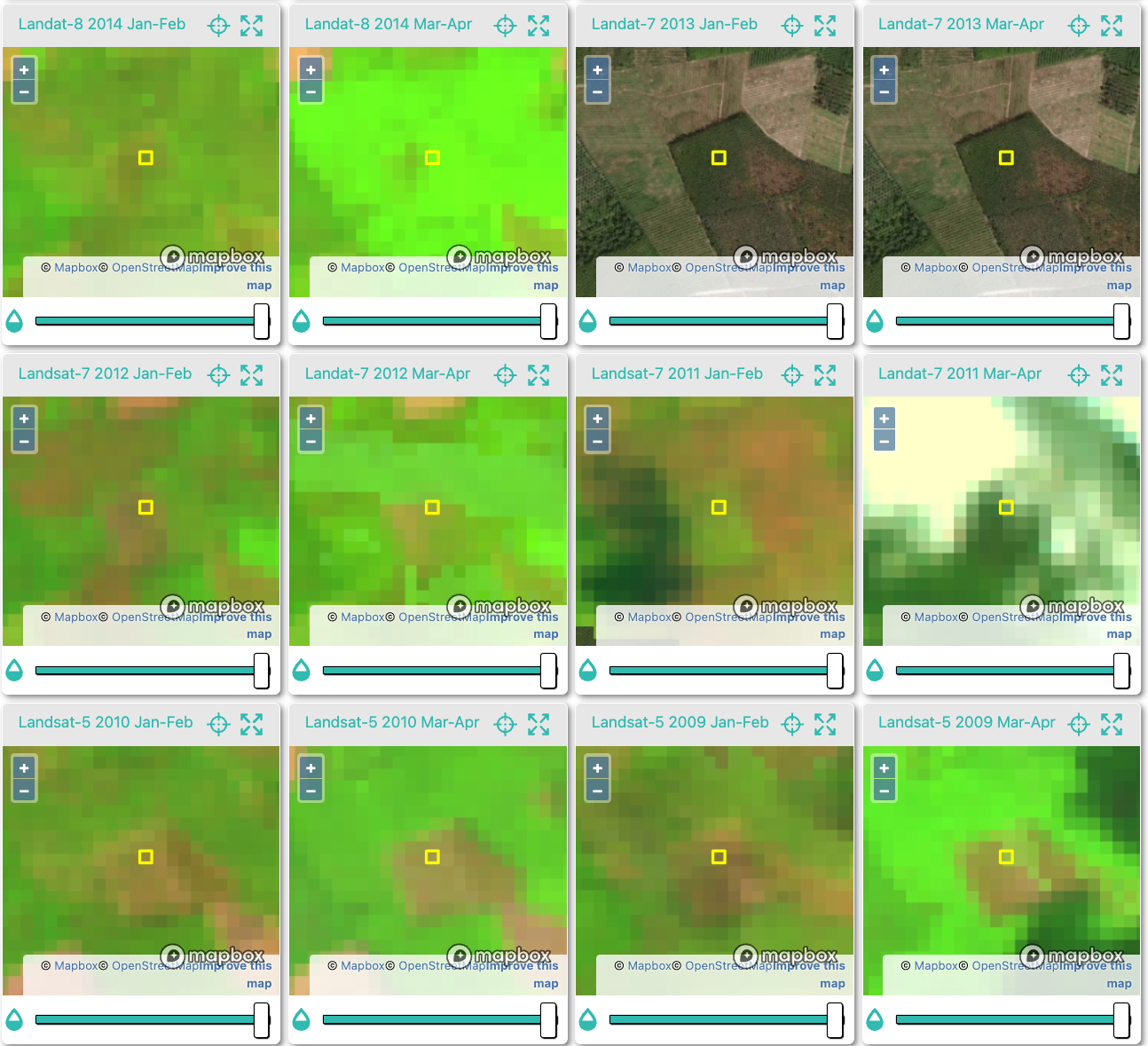 |
| 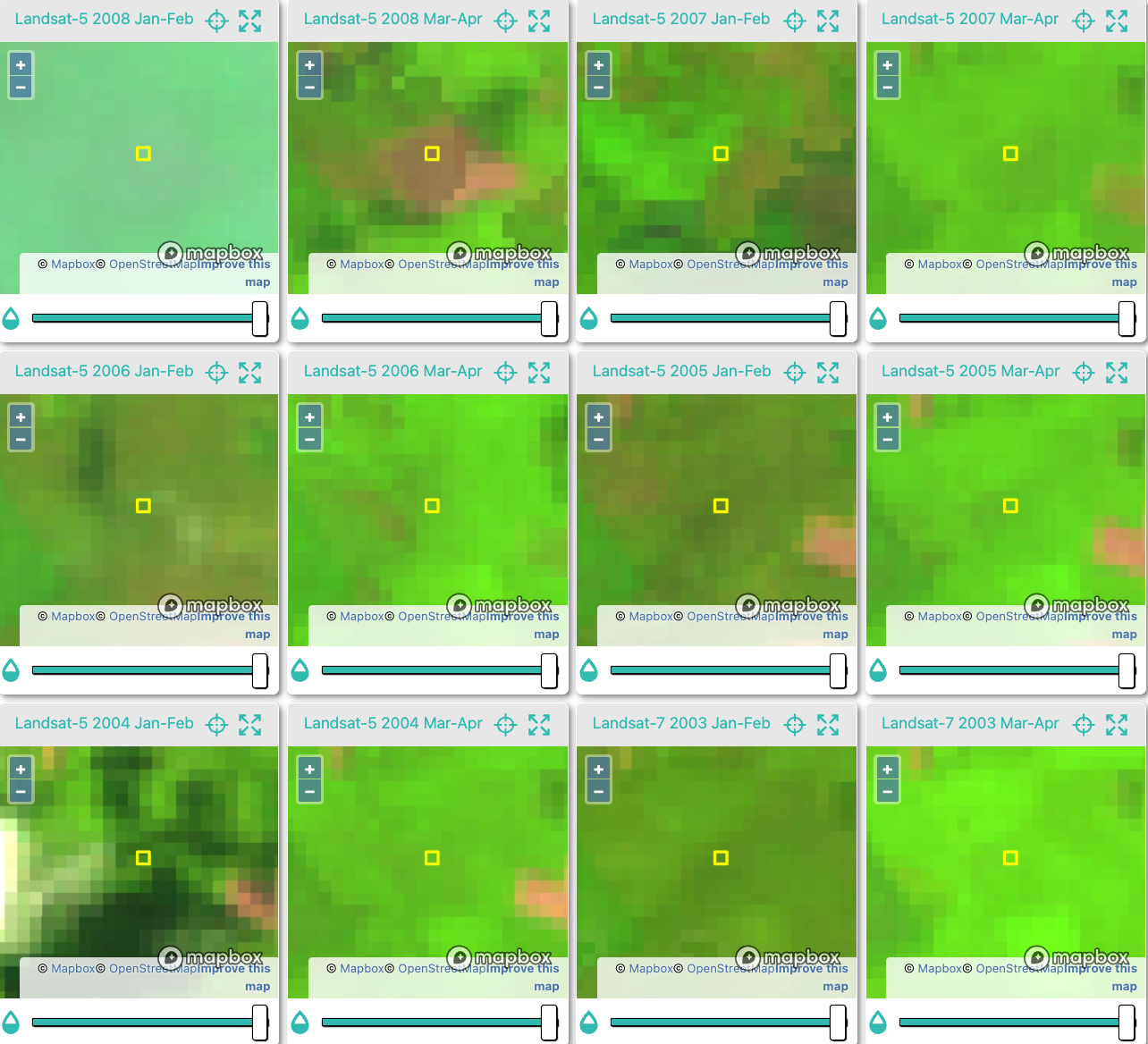 |
| 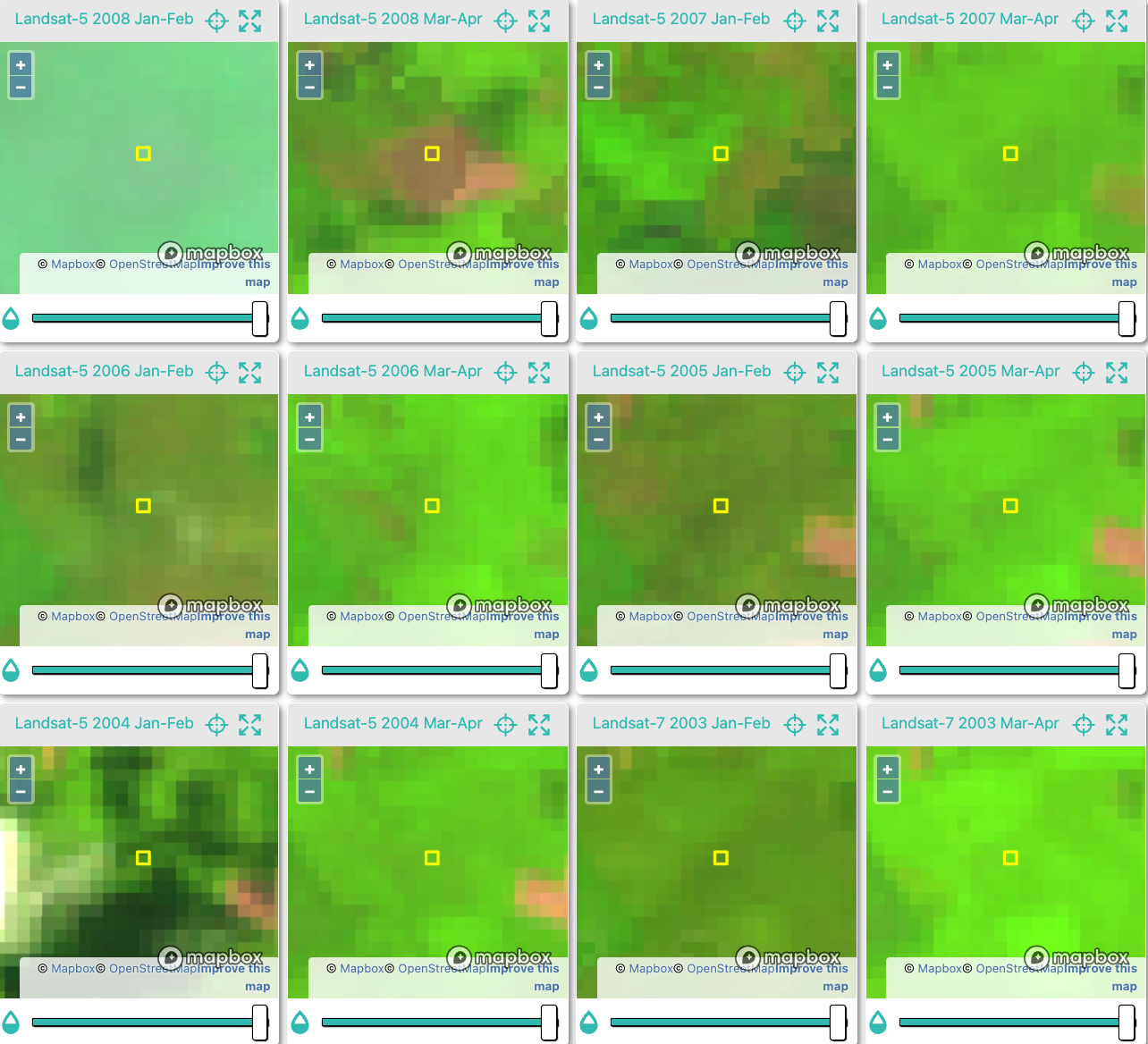 |
| 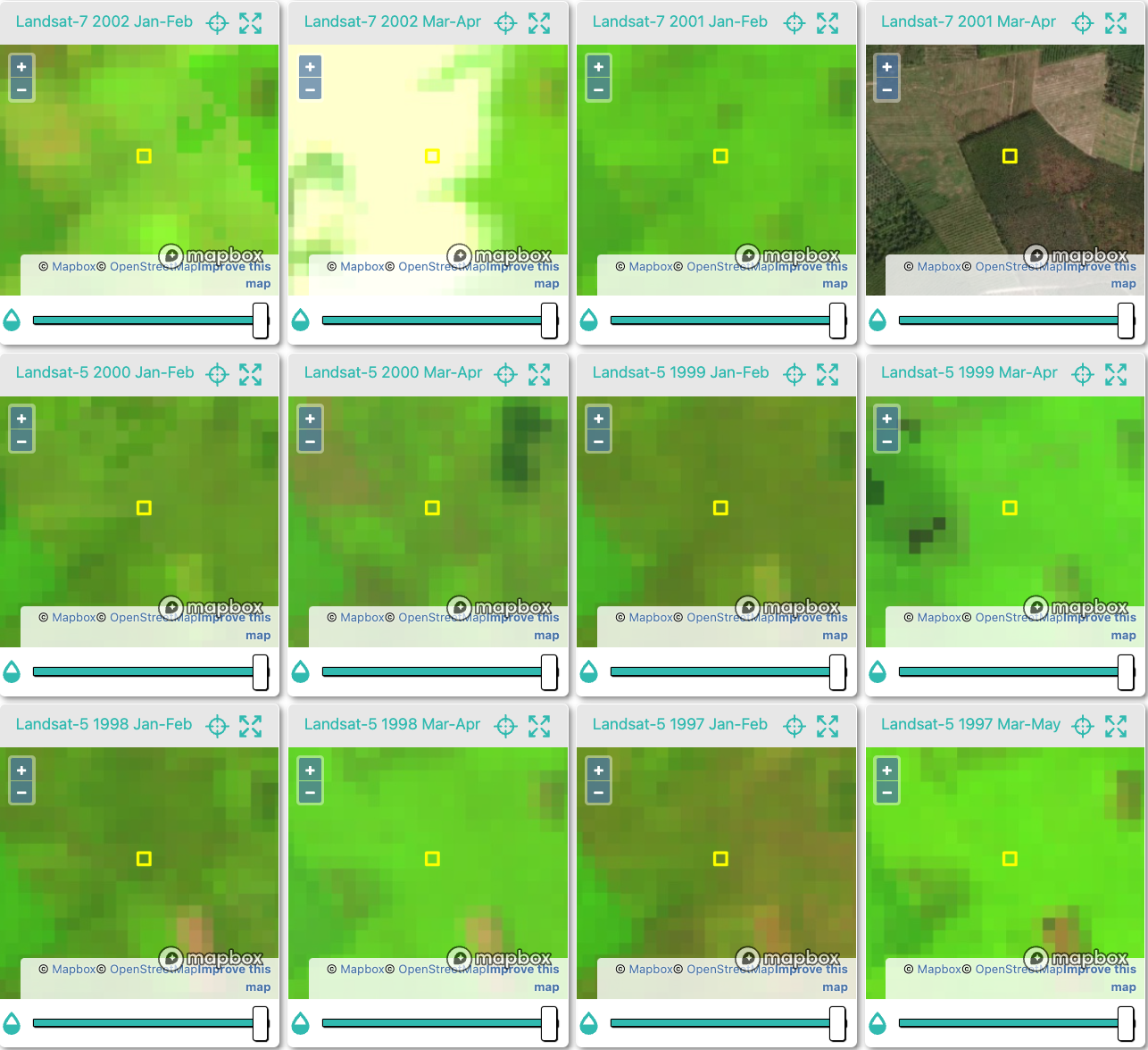 |
| 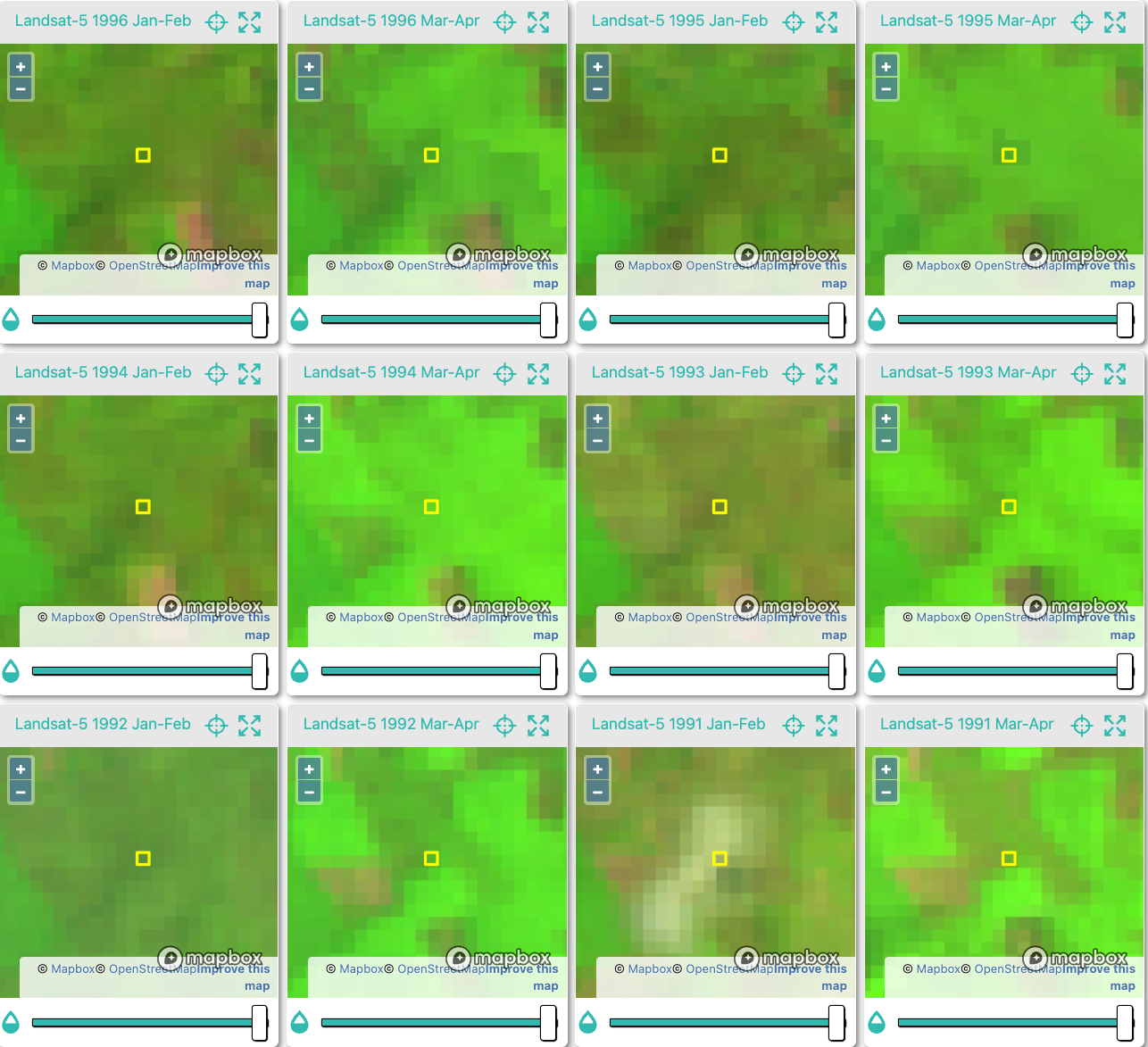 |
| 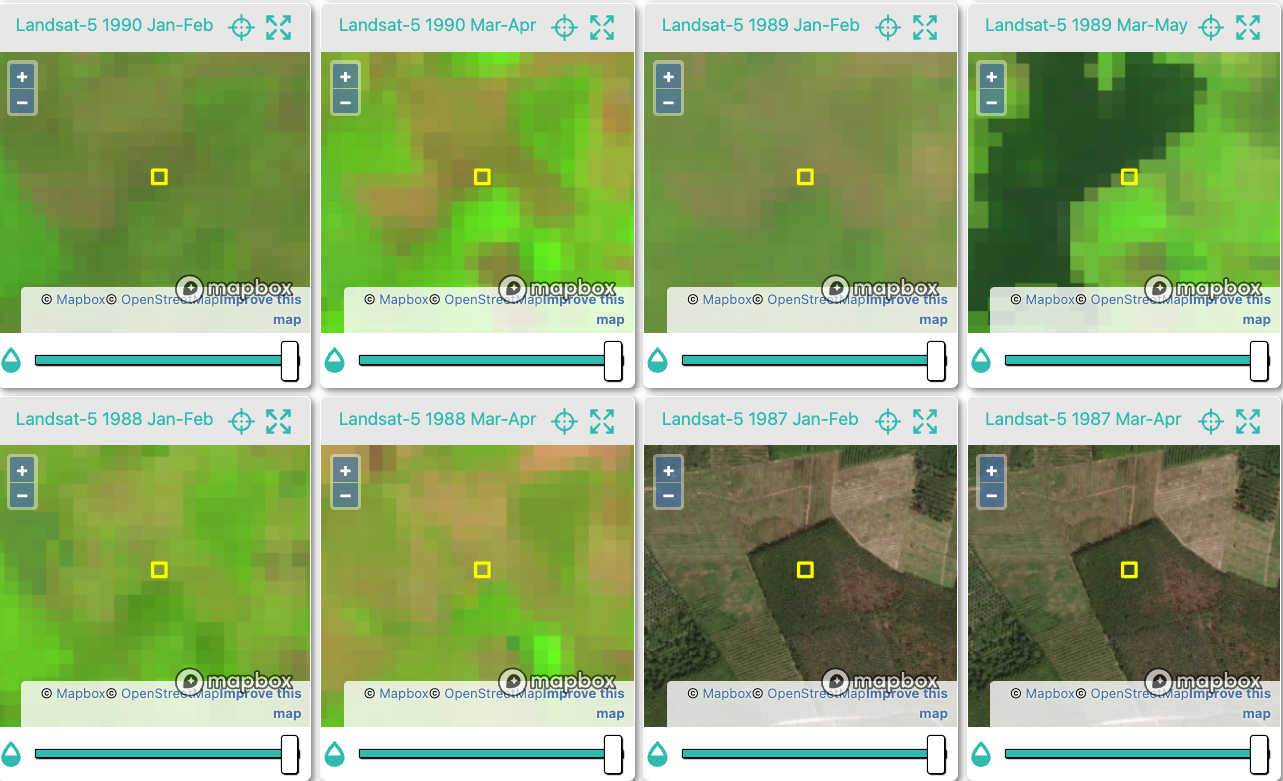 |
