# Supplementary material for: High-resolution maps show that rubber causes substantial deforestation
Source: Nature. 2023 Oct 18;623(7986):340–6. doi: 10.1038/s41586-023-06642-z (PMC10632130; doi:10.1038/s41586-023-06642-z)
Supplement: Supplementary file 2 — Reporting Summary [file 41586_2023_6642_MOESM2_ESM.pdf]

nature portfolio

Corresponding author(s): Antje Ahrends, Yunxia Wang

Last updated by author(s): Oct 10, 2023

Reporting Summary

Nature Portfolio wishes to improve the reproducibility of the work that we publish. This form provides structure for consistency and transparency in reporting. For further information on Nature Portfolio policies, see our Editorial Policies and the Editorial Policy Checklist.

Statistics

For all statistical analyses, confirm that the following items are present in the figure legend, table legend, main text, or Methods section.

n/a	Confirmed	
☐	☒	The exact sample size (*n*) for each experimental group/condition, given as a discrete number and unit of measurement
☐	☒	A statement on whether measurements were taken from distinct samples or whether the same sample was measured repeatedly
☐	☒	The statistical test(s) used AND whether they are one- or two-sided<br>*Only common tests should be described solely by name; describe more complex techniques in the Methods section.*
☐	☒	A description of all covariates tested
☐	☒	A description of any assumptions or corrections, such as tests of normality and adjustment for multiple comparisons
☐	☒	A full description of the statistical parameters including central tendency (e.g. means) or other basic estimates (e.g. regression coefficient) AND variation (e.g. standard deviation) or associated estimates of uncertainty (e.g. confidence intervals)
☒	☐	For null hypothesis testing, the test statistic (e.g. *F*, *t*, *r*) with confidence intervals, effect sizes, degrees of freedom and *P* value noted<br>*Give P values as exact values whenever suitable.*
☒	☐	For Bayesian analysis, information on the choice of priors and Markov chain Monte Carlo settings
☒	☐	For hierarchical and complex designs, identification of the appropriate level for tests and full reporting of outcomes
☒	☐	Estimates of effect sizes (e.g. Cohen's *d*, Pearson's *r*), indicating how they were calculated

Our web collection on statistics for biologists contains articles on many of the points above.

Software and code

Policy information about availability of computer code

Data collection	All software used for data collection (Google Earth Engine, Google Earth Pro, and Collect Earth Online) are publicly available.
Data analysis	All software used for data analysis (Google Earth Engine, and R 4.2.2) are publicly available and all code is deposited in a public repository: https://earthengine.googlesource.com/users/wangyxtina/Nature_rubber. Users with a Google Earth Engine account can access the code on: https://code.earthengine.google.com/?accept_repo=users/wangyxtina/Nature_rubber

For manuscripts utilizing custom algorithms or software that are central to the research but not yet described in published literature, software must be made available to editors and reviewers. We strongly encourage code deposition in a community repository (e.g. GitHub). See the Nature Portfolio guidelines for submitting code & software for further information.

Data

Policy information about availability of data

All manuscripts must include a data availability statement. This statement should provide the following information, where applicable:

- Accession codes, unique identifiers, or web links for publicly available datasets
- A description of any restrictions on data availability
- For clinical datasets or third party data, please ensure that the statement adheres to our policy

The earth observation datasets that supported the findings of this study are publicly available (e.g., Google Earth Engine data catalogue). The rubber and associated deforestation maps produced here (Fig. 1a,b) are available from https://doi.org/10.5281/zenodo.8425153. They are also available from within Google Earth Engine:

Rubber:
https://code.earthengine.google.com/?asset=users/wangyxtina/MapRubberPaper/rForeRub202122_perc1585DifESAdist5pxPFfinal
Associated forest loss:
https://code.earthengine.google.com/?asset=users/wangyxtina/MapRubberPaper/rRubber30m202122_deforestationAPI20012016_preNBR600

Research involving human participants, their data, or biological material

Policy information about studies with human participants or human data. See also policy information about sex, gender (identity/presentation), and sexual orientation and race, ethnicity and racism.

Reporting on sex and gender	n/a
Reporting on race, ethnicity, or other socially relevant groupings	n/a
Population characteristics	n/a
Recruitment	n/a
Ethics oversight	n/a

Note that full information on the approval of the study protocol must also be provided in the manuscript.

Field-specific reporting

Please select the one below that is the best fit for your research. If you are not sure, read the appropriate sections before making your selection.

☐ Life sciences ☐ Behavioural & social sciences ☒ Ecological, evolutionary & environmental sciences

For a reference copy of the document with all sections, see nature.com/documents/nr-reporting-summary-flat.pdf

Ecological, evolutionary & environmental sciences study design

All studies must disclose on these points even when the disclosure is negative.

Study description	Analysis of remotely-sensed data to map rubber in 2021 and to map and quantify associated deforestation
Research sample	Reference ground data (in total >3,800 points) based on field observations, augmented with visually interpreted very-high resolution satellite data
Sampling strategy	Stratified random. Sample sizes were pre-determined based on a good practices protocol (Olofsson et al. 2014. Good practices for estimating area and assessing accuracy of land change. Remote Sensing of Environment 148, 42-57)
Data collection	Of the over 3,800 points, 2,000 were based on randomly sampled reference ground data collected by the World Agroforestry Centre in 2010, covering entire Southeast Asia and consisting of a mix of field data and visually interpreted very-high resolution satellite data. We updated the classification for these points for 2021 following a visual interpretation in Collect Earth Online and Google Earth Pro. The remainder (>1,800 points) were points from randomly sampled field data covering mainland Southeast Asia.
Timing and spatial scale	The maps cover 1993 to 2021. The spatial extent encompasses all Southeast Asia at pixel resolutions of 10 and 30 m
Data exclusions	No data were excluded
Reproducibility	All findings are reproducible and the code to reproduce the findings is available on GitHub. Where parameter choices affected the outcomes, the choices and their effects are clearly outlined in the manuscript.
Randomization	Reference ground data were randomly split into training and test data using a random number generator.
Blinding	All relevant analyses and data collections were completed and the code was debugged before the results were revealed.

Did the study involve field work? ☐ Yes ☒ No

Reporting for specific materials, systems and methods

We require information from authors about some types of materials, experimental systems and methods used in many studies. Here, indicate whether each material, system or method listed is relevant to your study. If you are not sure if a list item applies to your research, read the appropriate section before selecting a response.

Materials & experimental systems

n/a	Involved in the study
☒	☐ Antibodies
☒	☐ Eukaryotic cell lines
☒	☐ Palaeontology and archaeology
☒	☐ Animals and other organisms
☒	☐ Clinical data
☒	☐ Dual use research of concern
☒	☐ Plants

Methods

n/a	Involved in the study
☒	☐ ChIP-seq
☒	☐ Flow cytometry
☒	☐ MRI-based neuroimaging
